# Bibliometric analysis of neurite orientation dispersion and density imaging: research patterns, evolution, and frontier

**DOI:** 10.3389/fnins.2026.1806164

**Published:** 2026-04-29

**Authors:** Shenghan Wang, Chao Wang, Wenwen Song, Jiangnan Lin

**Affiliations:** 1 Department of Radiology, The First Affiliated Hospital of Zhejiang Chinese Medical University (Zhejiang Provincial Hospital of Chinese Medicine), Hangzhou, China; 2 The First School of Clinical Medicine, Zhejiang Chinese Medical University, Hangzhou, China

**Keywords:** bibliometric analysis, CiteSpace, neurite orientation dispersion and density imaging, visualization, VOSviewer

## Abstract

**Background:**

Neurite orientation dispersion and density imaging (NODDI), an emerging diffusion MRI technique for estimating the microstructural pathology of brain tissue in vivo, has attracted significant research interest. However, a systematic bibliometric analysis of this field remains unexamined. This study aims to perform a bibliometric analysis of the NODDI literature to explore the current research landscape, identify emerging trends, and provide insights for future investigations.

**Methods:**

NODDI-related publications were retrieved from the Web of Science (WOS) and Scopus databases during the period of 2012 to 2025. CiteSpace, VOSviewer, and Bibliometrix R package were used to generate visualization maps.

**Results:**

A total of 679 publications related to NODDI were identified from WOS, including 653 research articles and 26 review papers. 844 relevant publications were retrieved from the Scopus database. After 2012, the number of publications on NODDI increased rapidly. Sweden demonstrated the highest average citation per paper, while the United States contributed the largest number of publications. University College London was the most productive institution. Hui Zhang was identified as the most prolific author, while Alexander DC achieved the highest average citation count. *NeuroImage* was recognized as the leading journal in terms of publication frequency. Common keywords included “diffusion magnetic resonance imaging,” “NODDI,” “brain,” and “multiple sclerosis.” Recent studies show the research focus is shifting from methodological development to clinical application, especially in the field of neuropsychiatric disorders, and is being integrated with emerging methodologies such as Mendelian randomization.

**Conclusions:**

This bibliometric analysis highlights potential directions for future NODDI-related research. Future studies may focus on optimizing imaging techniques, investigating neuropsychiatric disorders, and integrating advanced methodologies.

## Introduction

Diffusion magnetic resonance imaging (dMRI) is a crucial non-invasive technique for detecting tissue microstructure (Le Bihan and Johansen-Berg, 2012). It has been widely applied in the assessment of neurodegenerative diseases (Pasquini et al., 2022; Kamagata et al., 2020), psychiatric disorders (Kraguljac et al., 2023; Maziero et al., 2021), and white matter lesions (Zhao et al., 2021; Mustafi et al., 2019). Currently, diffusion tensor imaging (DTI) remains the standard clinical dMRI technique (Basser et al., 1994). Although DTI can sensitively detect microstructural changes through fractional anisotropy (FA) and mean diffusivity (MD), these inherently non-specific biomarkers complicate the precise identification of specific histological alterations, as variations in their values may stem from multiple pathological mechanisms including reduced neurite density, increased orientation dispersion, edema, or gliosis (Zhang et al., 2012; Andica et al., 2020). In response to these limitations, neurite orientation dispersion and density imaging (NODDI) were introduced by (Zhang et al. 2012), marking a significant advancement in the field. As a representative of multi-shell dMRI technology, NODDI achieves specific quantitative characterization of the microstructural organization of the entire brain through three core scalar parameters: Neurite Density Index (NDI), Orientation Dispersion Index (ODI), and Isotropic Volume Fraction (ISOVF; Zhang et al., 2012; Li et al., 2024; Kamiya et al., 2020).

With its unique ability to quantify neurite density and orientational spread, NODDI has shown promising results in both clinical and basic research. It has been widely used in neurology, psychiatry and oncology, and plays an important role in diagnosis, disease progression monitoring and pathological mechanism exploration. In neurology, NODDI can accurately capture the microstructure changes of the brain in patients with Alzheimer's disease, Parkinson's disease and multiple sclerosis, and provide reliable imaging evidence for early identification and differential diagnosis of diseases (Parker et al., 2018; Mitchell et al., 2019; Seyedmirzaei et al., 2023). In psychiatry, it has effectively revealed the brain microstructural abnormalities associated with schizophrenia, major depression, and autism spectrum disorder, thus deepening the understanding of the neurobiological basis of these psychiatric disorders (Ota et al., 2018; Szeszko et al., 2025; Andica et al., 2021). In oncology, NODDI has also shown important application value. By quantifying the microstructural changes in tumor parenchyma and surrounding areas, it can help to distinguish gliomas from solitary brain metastases and predict the grade and cell proliferation of meningiomas (Bai et al., 2024; She et al., 2023). Apart from clinical applications, NODDI has also been increasingly used in basic neuroscience research, which has promoted the in-depth study of brain development, aging process and neural plasticity mechanisms (Wang et al., 2019; Colon-Perez et al., 2019; Han et al., 2024).

Given the rapid global adoption of NODDI and its expanding clinical applications (Seyedmirzaei et al., 2023; Naval-Baudin and Pons-Escoda, 2024; Pillai et al., 2025), there is an urgent need to map the current research landscape of this technology systematically and quantitatively. Bibliometric analysis, through the integration of statistical and visualization techniques, enables quantitative evaluation of literature within specific research domains and reveals the developmental patterns and trends of scientific topics (Ninkov et al., 2022). Compared to traditional narrative reviews, this approach provides a visual mapping of the relationships among authors, institutions, countries, journals, and keywords, thereby offering a clearer overview of the research landscape and emerging trends (Chen and Song, 2019). Conducting a bibliometric analysis of NODDI can deepen the understanding of the current research status of this technology, identify the gaps in the current research, and further optimize its application direction. Therefore, this study aimed to systematically evaluate the development trajectory, current research landscape and future trends in the field of NODDI through bibliometric analysis, and ultimately provide theoretical reference and actionable insights for researchers to determine future research directions. We present this article in accordance with the BIBLIO reporting checklist (Montazeri et al., 2023).

## Materials and methods

### Data source and retrieval strategies

A comprehensive literature search was conducted in the Web of Science (WoS) and Scopus databases on December 31, 2025. For the WoS database, the search query was defined as follows: (((TI=(“NODDI” OR “Neurite Orientation Dispersion and Density Imaging” OR “Neurite Density Index” OR “Orientation Dispersion Index”)) OR AB=(“NODDI” OR “Neurite Orientation Dispersion and Density Imaging” OR “Neurite Density Index” OR “Orientation Dispersion Index”)) OR AK=(“NODDI” OR “Neurite Orientation Dispersion and Density Imaging” OR “Neurite Density Index” OR “Orientation Dispersion Index”) AND DOP = (2012-01-01/2025-12-31)) AND DT = (Article OR Review) AND LA = (English). For the Scopus database, the retrieval strategy was formulated as: TITLE-ABS-KEY (“NODDI” OR “Neurite Orientation Dispersion and Density Imaging” OR “Neurite Density Index” OR “Orientation Dispersion Index”) AND PUBYEAR > 2011 AND PUBYEAR < 2026 AND (LIMIT-TO (DOCTYPE, “ar”) OR LIMIT-TO (DOCTYPE, “re”)) AND (LIMIT-TO (LANGUAGE, “English”)).

Two independent researchers screened publications to minimize bias and exclude non-relevant records. A third researcher resolved discrepancies through consensus. Strict inclusion and exclusion criteria were formulated for literature screening: inclusion criteria refer to English peer-reviewed original articles and reviews focusing on NODDI or its core parameters, which were published in 2012–2025 and indexed in WoS or Scopus; exclusion criteria include non-English publications, non-article/review document types, NODDI-irrelevant studies, intra-database duplicate records, and publications with inaccessible full text or abstracts. Methodological quality was assessed by three researchers using the BIBLIO checklist.

**Figure 1:**
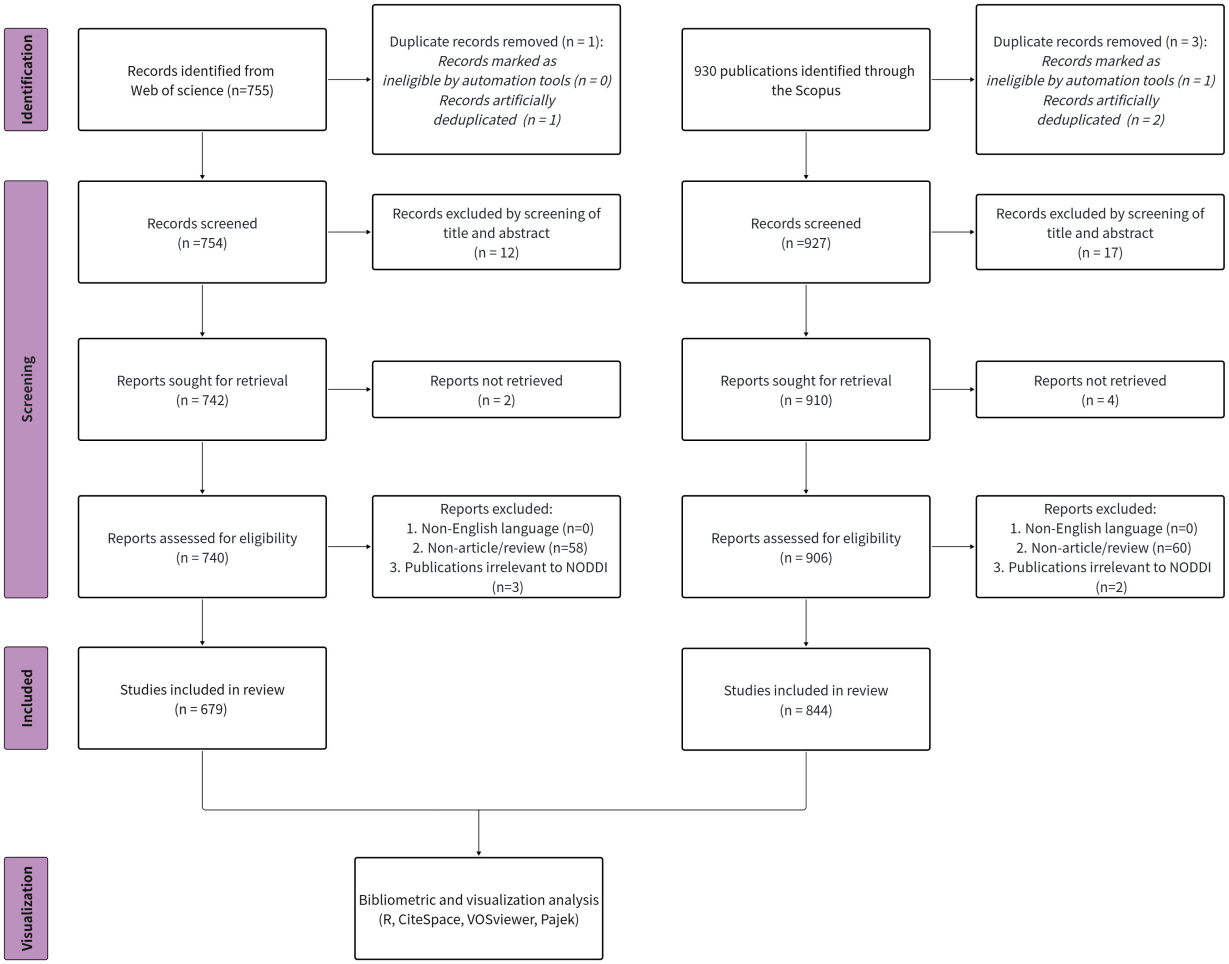
Flowchart of the search strategy in the study.

The extracted dataset comprised publication title, keywords, authors, affiliations, country/region, abstract, and publication date. A detailed flowchart of the study selection process is provided (Figure 1).

### Bibliometric indicators

Multiple bibliometric indicators were used in this study. The dynamic evolution of the study was tracked by the number of publications. The academic influence was evaluated objectively by the citation frequency. The h/g/m index system was used to quantify the performance of researchers and institutions. The mechanism and keyword co-occurrence network were analyzed based on frequency statistics and centrality measurement (node centrality threshold ≥0.1). To identify short-term academic hotspots by citation burst detection. The evolution of research hotspots was revealed by reference and keyword clustering. Modularity index (*Q* > 0.3 to confirm the statistical significance of clusters) and contour coefficient (*S* > 0.5 to indicate the quality of clusters, *S* > 0.7 to indicate high-reliability clusters) were used to verify the validity of the analysis results.

### Data analysis and visualization

Given the intrinsic variations in data architectures between WoS and Scopus, unmediated integration of the two datasets would incur the loss of critical information. For this reason, the two databases were analyzed separately to safeguard the robustness of the study results. It is worth noting that, in view of the scholarly quality and indexing authority of WoS, all subsequent core analysis were predominantly based on its dataset. Analysis derived from Scopus data, encompassing annual publication trends and keyword clustering, are presented in the Supplementary material for reference.

Data processing and visualization were performed using R software (version 4.4.1), CiteSpace (version 6.4.R1 Advanced), VOSviewer (version 1.6.20), and Pajek (version 6.01).

The bibliometric data obtained from CiteSpace and the Bibliometrix R package were analyzed in R software to generate statistical visualizations. Publication volume trends exported from CiteSpace were fitted using polynomial regression models in Excel to forecast future research trajectories.

CiteSpace was utilized to construct a co-occurrence network of institutions, co-cited literature and keywords, as well as a time axis visualization and a keyword burst detection graph (Chen, 2006).

Bibliometrix R was employed to generate thematic trend charts for authors, journals, and keywords (Aria and Cuccurullo, 2017).

Additionally, collaboration networks among countries/regions, authors and journals were constructed using the association strength algorithm in VOSviewer and further refined in Pajek to optimize network structure (van Eck and Waltman, 2010).

## Results

### Overall publication trend and literature distribution

**Figure 2:**
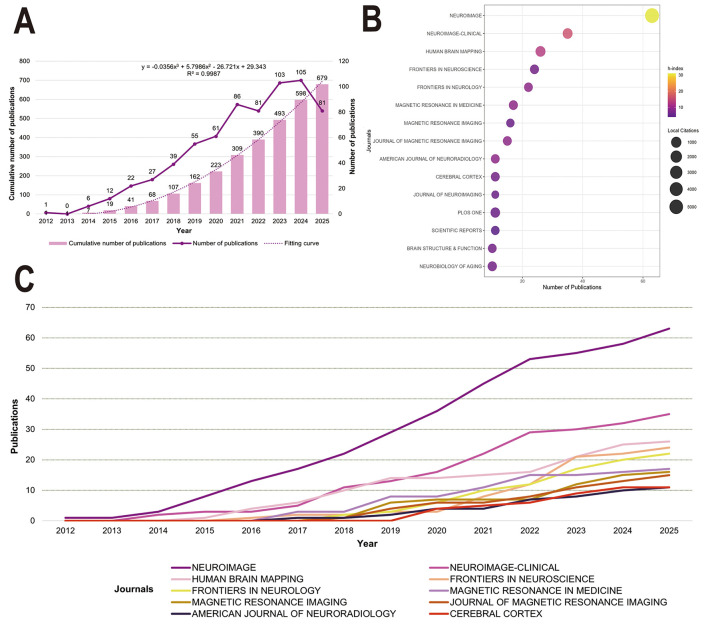
Overall publication trends and literature distribution visualization. **(A)** Temporal evolution of research publications in the field of NODDI. **(B)** Bubble plot of the top 15 journals by number of publications. The vertical axis marks the journal name, and the horizontal axis represents the number of publications. The bubble size maps the total citation frequency, and the color gradient reflects the journal h-index. **(C)** Evolution trend of annual publication volume of top 10 journals.

A total of 679 publications related to NODDI were identified from WoS, including 653 research articles (96.2%) and 26 reviews (3.8%). The field has experienced steady growth since its first publication in 2012 (Figure 2A). To predict future research trends, a cubic polynomial regression model was fitted to historical cumulative publication data (Figure 2A, dashed line). The model demonstrated an excellent fit with observed data from 2012 to 2025 (*R*^2^ = 0.9987), supporting its predictive validity. Based on the model, the cumulative number of NODDI-related publications is projected to reach about 1,358 by 2030. The prediction is based on the historical growth pattern, and the actual growth in the future may be affected by factors such as technological breakthroughs and the speed of clinical translation. Following duplicate removal, 844 unique records were extracted from Scopus. The temporal publication trend was consistent with that of WoS (Supplementary Figure S1), reflecting a steadily growing research focus on NODDI.

The journal influence analysis revealed (Figure 2B) that *NeuroImage* led in both output and influence, ranking first in the number of publications (*n* = 63), H-index (*h* = 31) and total citations (5,585). This was followed by *NeuroImage: Clinical* (*n* = 35, h = 16), which was ranked second with 744 citations. Other notable journals included *Human Brain Mapping* (783 citations) and *Plos One* (541 citations). Supplementary Table S1 provides the top 20 composite measures.

The temporal trend of journal output (Figure 2C) indicated that *NeuroImage* has consistently published the highest number of NODDI-related studies. Since 2020, *NeuroImage: Clinical* has exhibited the most rapid growth in annual publication volume, making it the fastest expanding journal in the field after *NeuroImage*.

### Analysis of countries and institutions collaboration networks

**Figure 3:**
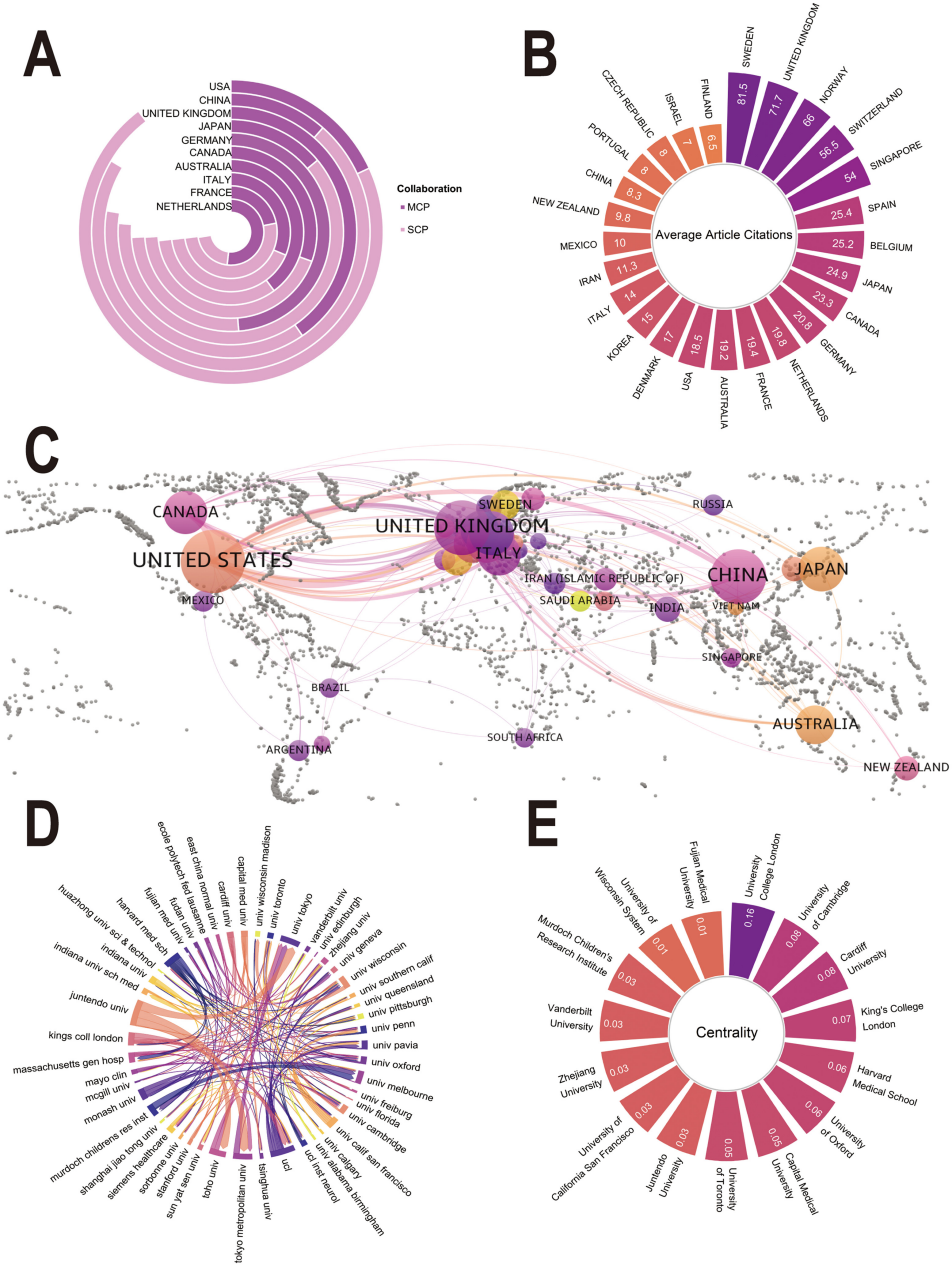
Countries/regions and institutions of publications in the field of NODDI. **(A)** Countries/regions of publication. Classification by single national publication (SCP) and multiple national publication (MCP). **(B)** Top 25 most cited countries/regions. **(C)** Map of countries/regions cooperation networks. Nodes represent each country/region, the size of nodes reflects the number of publications, and links indicate the strength of collaboration. **(D)** Collaboration chord diagram of institutions. The lines connecting nodes represent collaboration or co-occurrence relationships. **(E)** The centrality of the top 15 most productive institutions.

Analysis of the corresponding author countries revealed distinct global contributions and collaboration patterns (Supplementary Table S2). The United States led in publication output (*n* = 193, 28.4%), followed by China (*n* = 138, 20.3%), the United Kingdom (*n* = 77, 11.3%), Japan (*n* = 60, 8.8%), and Germany (*n* = 35, 5.2%). China and Japan showed a greater focus on domestic research, with the highest proportion of single national publications (SCP: 86.2% and 81.6%). In contrast, European countries showed a stronger tendency for international collaboration, with the Netherlands having the highest proportion of multinational publications (MCP: 71.4%), followed by Germany (65.7%; Figure 3A).

The volume of publications at the country level did not directly correspond to academic impact (Figure 3B). Sweden received the highest average number of citations per paper (81.5), followed by the United Kingdom (71.7). Although the United States published the largest number of papers, it ranked 14th in average citation (18.5). This may be related to the large volume of publications in the United States, which cover a large number of early exploratory or methodological validation studies, whose citation frequency is typically lower than that of mature clinical application studies. The geographical collaboration network further highlighted the central role of the United Kingdom, which showed the highest total link strength (TLS =179), indicating extensive international partnerships in the European and Asian regions (Figure 3C). The United States ranked second in this category (TLS = 176). Detailed productivity and citation indicators at the country level are presented in Supplementary Tables S2, S3.

At the institutional level, collaborative network analysis revealed synergies among major research institutions (Figure 3D). Among these, Juntendo University has established particularly close cooperation links with Toho University, the University of Tokyo, and the Tokyo Metropolitan University. Among all institutions, University College London was the most productive with 64 publications and the highest centrality (0.16), indicating its role as a key hub in the global collaboration network (Figure 3E, Supplementary Table S4).

### Analysis of core authors and high-impact literature

**Figure 4:**
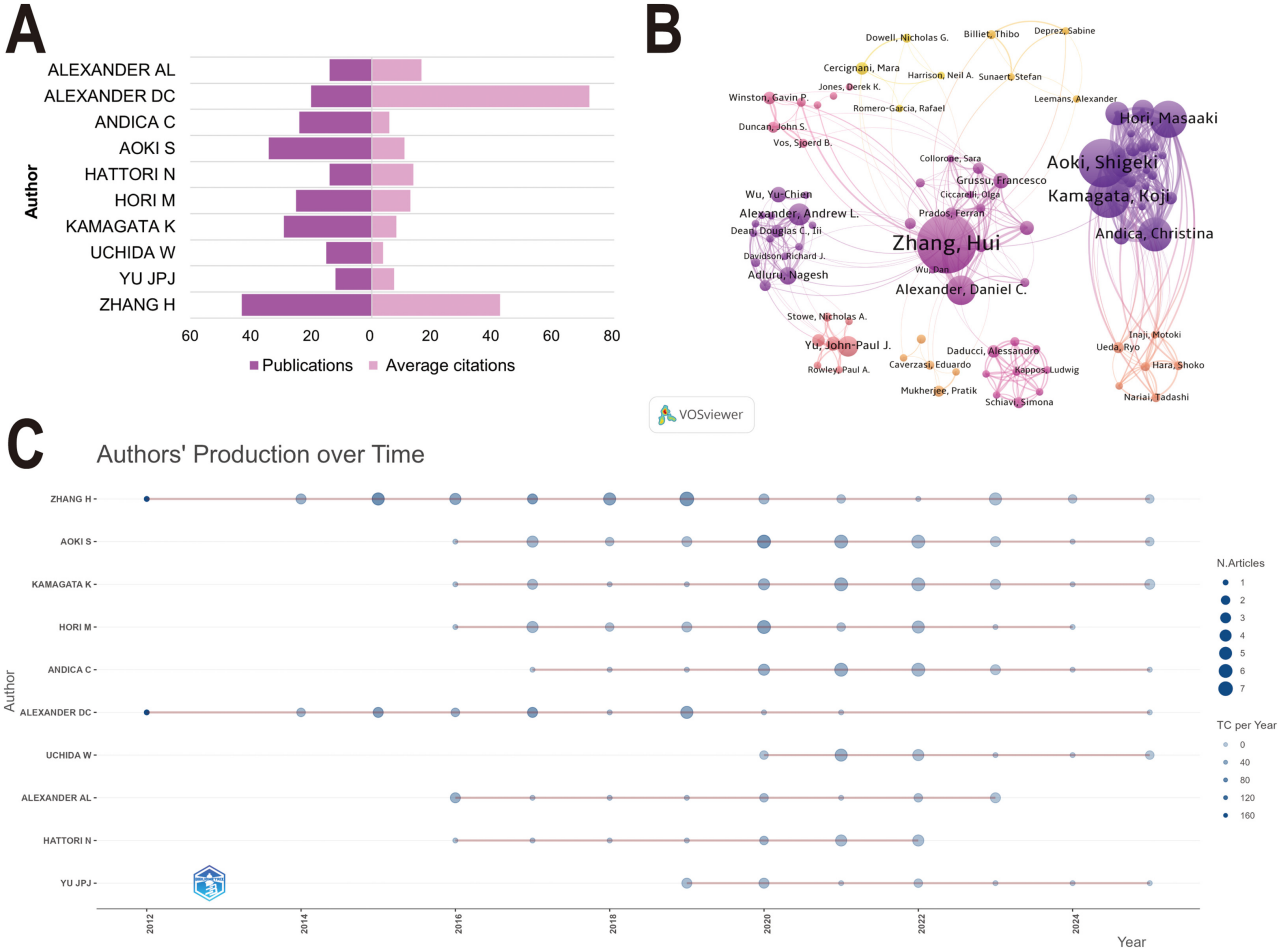
Visualization of the authors analysis. **(A)** The number of publications and average citations per article for the top 10 authors by productivity. **(B)** Co-occurrence network diagram of authors. Nodes represent different authors, with colors differentiating collaborative clusters. **(C)** Publication trends over time for the top 10 authors. Node size indicates the number of articles, while color intensity reflects total citation counts.

At the author level (Figure 4A), Zhang H dominated in output (43 publications) and impact, achieving the highest h-index (29), g-index (43), and m-index (2.07). Alexander DC had the highest average citations per paper (71.85 citations) and ranked second in h-index (18). The co-author network analysis identified five key research groups, led by Zhang H, Aoki S, Alexander AL, Daducci A, and Yu JPJ (Figure 4B). Within this collaborative landscape, Aoki S and Kamagata K emerged as core connection points, with the highest total link strength of 212 and 186, respectively (Supplementary Table S5). The evolving contributions of the top 10 authors are further illustrated by their publication outputs and annual citation trends (Figure 4C).

Analysis of the most cited publications highlights the fundamental importance of methodological innovation in the field (Table 1). In 2012, Zhang et al. published the seminal paper, “NODDI: Practical *in vivo* neurite orientation dispersion and density imaging of the human brain,” which was the most cited study (*n* = 2,288). It was followed by the 2015 study by Daducci et al., “Accelerated Microstructure Imaging via Convex Optimization (AMICO) from diffusion MRI data” (*n* = 382).

**Table 1:** List of the top 10 most cited publications.

Rank	Author	Journal	Publication year	Total citations	TC per year	DOI
1	Zhang H	Neuroimage	2012	2288	163.43	doi: 10.1016/j.neuroimage.2012.03.072
2	Daducci A	Neuroimage	2015	382	34.73	doi: 10.1016/j.neuroimage.2014.10.026
3	Cox SR	Nat Commun	2016	325	32.50	doi: 10.1038/ncomms13629
4	Grussu F	Ann Clin Transl Neur	2017	226	25.11	doi: 10.1002/acn3.445
5	Golkov V	Ieee T Med Imaging	2016	207	20.70	doi: 10.1109/TMI.2016.2551324
6	Kamiya K	J Neurosci Meth	2020	184	30.67	doi: 10.1016/j.jneumeth.2020.108908
7	Batalle D	Neuroimage	2017	175	19.44	doi: 10.1016/j.neuroimage.2017.01.065
8	Lampinen B	Neuroimage	2017	163	18.11	doi: 10.1016/j.neuroimage.2016.11.053
9	Colgan N	Neuroimage	2016	159	15.90	doi: 10.1016/j.neuroimage.2015.10.043
10	Billiet T	Neurobiol Aging	2015	158	14.36	doi: 10.1016/j.neurobiolaging.2015.02.029

### Research hotspots and theme evolution

**Figure 5:**
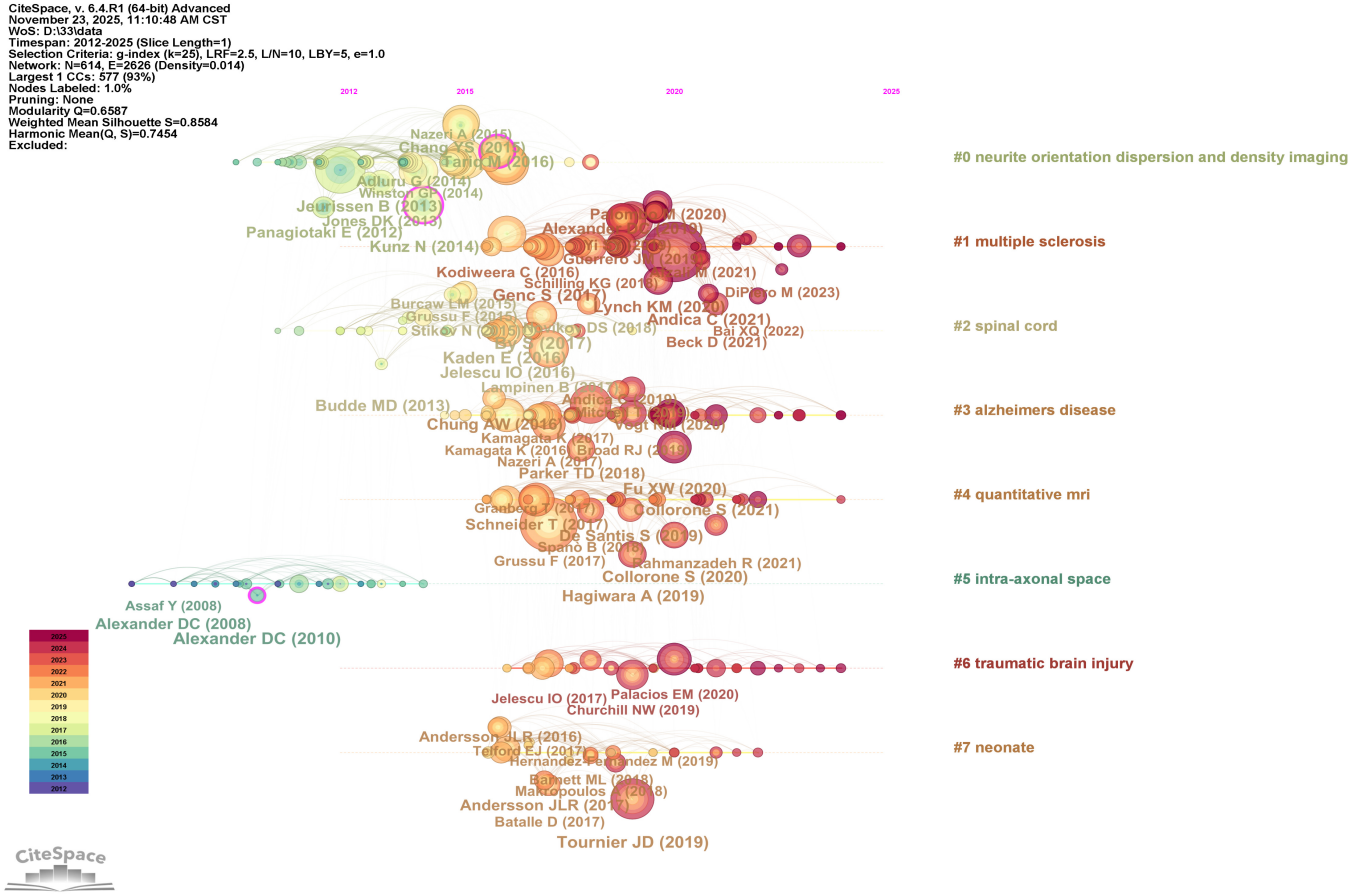
Timeline view of the cited references. The cluster tag on the right shows the topic. The larger the node, the higher the citation frequency of the literature. The horizontal axis position of the node only indicates the year when the document was first cited.

In order to reveal the research hotspots and topic evolution in the field of NODDI, we combined the literature co-citation and key words analysis. The timeline visualization of co-citation clusters identified eight major themes (Figure 5), including “neurite orientation dispersion and density imaging,” “multiple sclerosis,” “spinal cord,” “Alzheimer's disease,” “quantitative MRI,” “intra-axonal space,” “traumatic brain injury,” and “neonate.” This analysis illustrates the research trajectory of this field, starting from the development of methodology, and gradually expanding to specific diseases and anatomical regions.

To further expand the analytical dimensions, this study used VOSviewer to extract 5,551 keywords from the Scopus database (Supplementary Figure S2). Keyword co-occurrence analysis revealed four key research clusters in the NODDI domain: MRI-based human neuroimaging and pathology studies, population demographic and clinical characteristic profiling, diffusion-weighted imaging technology and neural mechanism research, and diffusion tensor imaging and its clinical applications.

**Figure 6:**
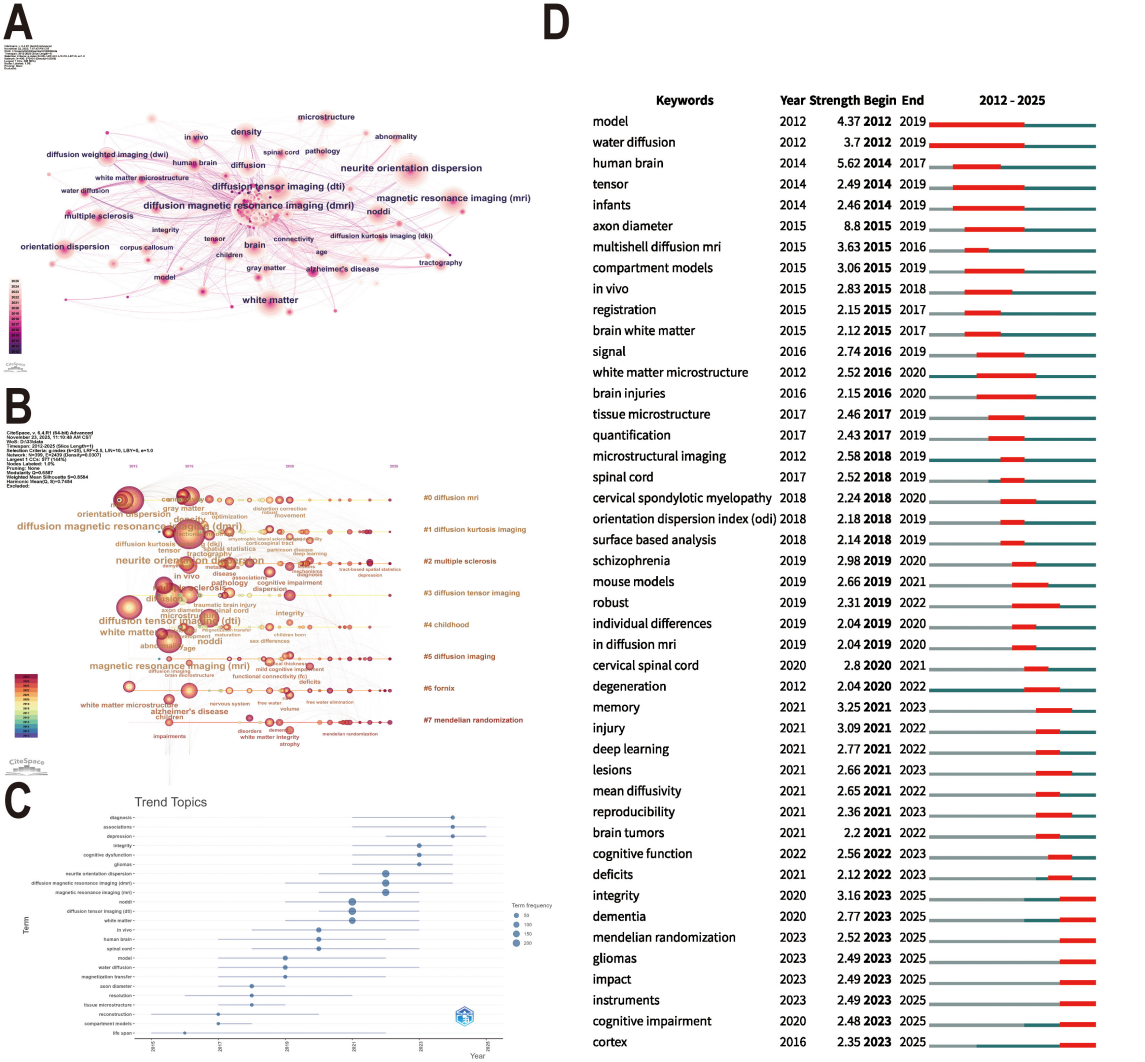
Visual analysis of keywords and topics. **(A)** Keywords co-occurrence network diagram. Nodes represent research keywords, with their size reflecting frequency of appearance; larger nodes indicate higher frequency. The color bands around the nodes represent the time span. Nodes with a purple border indicate centrality ≥ 0.1. The lines connecting nodes represent collaboration or co-occurrence relationships. **(B)** Timeline view of keywords. **(C)** Trending topics for keywords from 2012 to 2025. **(D)** The top 45 keywords with the strongest citation bursts. The timeline is depicted as a blue line, and the red segment is the burst time slot on the blue timeline.

An in-depth analysis of keywords further refined the above findings. “Neurite orientation dispersion” (205 occurrences), “diffusion magnetic resonance imaging (dMRI)” (185 occurrences) and “diffusion tensor imaging (DTI)” (154 occurrences) was the most frequent keywords in the field, which confirmed the field's core focus on advanced imaging techniques (Figure 6A, Supplementary Table S6). The keyword timeline analysis identified eight core themes, dMRI, diffusion kurtosis imaging (DKI), multiple sclerosis, DTI, childhood, diffusion imaging, fornix, and Mendelian randomization (MR). Among them, the research heat of traditional technology topics such as dMRI and DTI has remained stable, while Mendelian randomization and multiple sclerosis represent the active research fronts in recent years (Figure 6B).

The topic evolution diagram (Figure 6C) further reveals the shift of research focus from the time dimension. Early research (2015–2019) focused on the exploration of microstructure parameters such as “axon diameter” (burst strength = 8.8, Figure 6D). In recent years, research focus has shifted to the application of NODDI in disease diagnosis and mechanism analysis. In particular, between 2023 and 2025, there was a sudden growth in the use of keywords such as “integrity,” “dementia,” “Mendelian randomization,” “glioma,” “impact,” “tool,” “cognitive impairment,” and “cortex.” This suggests that the field is moving toward the frontier of neurodegenerative disease mechanisms, tumor microenvironment, genetic causal inference, and cognitive dysfunction.

## Discussion

This study firstly quantitatively analyzed NODDI literature from 2012 to 2025 using bibliometric methods. The number of publications in the field of NODDI has risen from a single article in 2012 to 679 articles to date. This study highlights the clinical applications and evolving trends of this technology, offering a reference for future research.

There are notable regional disparities in the international collaboration patterns of NODDI research. European countries, including the Netherlands (MCP: 71.4%), Germany (MCP: 65.7%), and the United Kingdom (MCP: 51.9%), exhibit a significantly higher proportion of internationally co-authored publications compared to Asian counterparts such as China (MCP: 13.8%) and Japan (MCP: 18.3%; Supplementary Table S2). This discrepancy may be attributed to the geographical proximity within Europe, a well-coordinated research infrastructure, and a relatively harmonized policy framework for data sharing (Kondylakis et al., 2023). The Horizon Europe framework initiative's promotion of cross-border collaboration, alongside the establishment of large-scale domestic cohorts in Asian countries (e.g., CHIMGEN). Existing studies have demonstrated that publications involving cross-institutional or multinational collaborations tend to achieve higher impact factors (Le Grand et al., 2024; Mak et al., 2024; Cox et al., 2016). This may potentially be due to the integration of diverse imaging datasets in international collaboration and the cross-validation and innovation of methodologies. Nevertheless, despite its advantages, international collaboration encounters several challenges, particularly technical barriers, legal complexities, and insufficient incentive mechanisms related to data sharing (Giehl et al., 2024). This is especially prominent in the process of integrating data across multiple centers and different ethnic groups. Therefore, future international collaboration should focus on enhancing the universality of data models while deepening the interpretation of population-specific characteristics across different regions. Both aspects are crucial for promoting the global application of NODDI technology.

The top ten most-cited papers in the NODDI field (Table 1) have had a significant impact on the methodological development, computational optimization, and clinical applications of this technique. (Zhang et al. 2012) introduced the NODDI model framework for the first time, establishing the theoretical foundation for NODDI in neuroimaging (cited 2,288 times). Subsequently, (Daducci et al. 2015) enhanced computational efficiency by developing the AMICO framework. (Golkov et al. 2016) incorporated deep learning solutions, driving model innovation. (Batalle et al. 2017) further expanded the technical dimension by integrating brain network features. In terms of key technical applications, (Cox et al. 2016) revealed the genetic association between age-related white matter microstructure and neurodegeneration. (Grussu et al. 2017) demonstrated that neurite-directed dispersion can serve as a novel imaging marker for multiple sclerosis. (Colgan et al. 2016) confirmed that NODDI outperforms DTI in histological specificity for tau pathology in Alzheimer's disease. Collectively, these core achievements have built a crucial bridge for NODDI technology to advance from theoretical models to animal experimental validation and further to clinical practice. Along with technological progress, this field has shown a clear trend toward clinical translation. Animal experiments represent a key link in technological translation, serving as an important connection between basic research and clinical practice. Accordingly, NODDI has been widely applied in various animal model studies and investigations of brain microstructural alterations in diverse neurological disorders (Gatto et al., 2018, 2019, 2021).

In terms of scientific journals, *NeuroImage* emerged as the most prominent source, reflecting its high academic standing and influence within the neuroimaging community (Figure 2B). According to the 2025 Journal Citation Reports (JCR), the journal occupies a Q1 position in the Neuroimaging subcategory and holds an impact factor of 4.5. This leading position was further confirmed in the analysis of highly cited articles (Table 1). Five of the top ten most cited articles were published in *NeuroImage* (Zhang et al., 2012; Daducci et al., 2015; Batalle et al., 2017; Colgan et al., 2016; Lampinen et al., 2017). Furthermore, *NeuroImage* has consistently led in the volume of NODDI-related publications since 2012, demonstrating a steady growth in annual output (Figure 2C). *NeuroImage* serves as a premier platform for research on imaging theory and technological innovation, featuring methodological advances in multi-modal neuroimaging for studying brain architecture and function. Its sister journal, *NeuroImage: Clinical*, ranks second in publication volume and also holds a JCR Q1 position in its subcategory, with a 2025 impact factor of 3.6. This journal specializes in neurological disorders and abnormalities, providing a dedicated forum for studies of structural and functional neural pathologies using imaging techniques. Based on their distinct editorial scopes, *NeuroImage* is recommended for studies emphasizing NODDI methodological innovations and technical refinements, while *NeuroImage: Clinical* is more appropriate for research exploring clinical applications of NODDI in disease diagnosis, progression monitoring, and treatment assessment.

The keyword time evolution map (Figure 6C) and emergence analysis (Figure 6D) clearly revealed the evolution path of NODDI research hotspots, showing a significant transition from spinal cord lesion to brain injury, and then to neuropsychiatric disorders. From 2018 to 2019, spinal cord has become an important field of clinical application of NODDI. (By et al. 2017) applied NODDI to cervical spinal cord assessment in patients with multiple sclerosis and found that this technique provided unique microstructure contrast. (Okita et al. 2018) further demonstrated that NODDI was superior to traditional DTI measures in predicting postoperative outcomes in patients with cervical spondylotic myelopathy. The histological validation study by (Grussu et al. 2017) provided key evidence for the biological validity of NODDI and found that its parameters were highly consistent with histological findings (*P* < 0.001). At the same time, the field of brain injury has also formed a research hotspot. A longitudinal study by (Palacios et al. 2020) showed that NODDI had superior sensitivity to detect axonal degeneration processes after mild traumatic brain injury (mTBI) compared with DTI. (Wu et al. 2018) further confirmed that NODDI showed an excellent ability to detect changes in white matter microstructure in the acute phase of mTBI.

In recent years, there has been a shift toward neuropsychiatric disorders (Figure 6C). A number of innovative studies have confirmed the importance of NODDI in the assessment of cognitive impairment and brain microstructure integrity. (Zhou et al. 2024) systematically analyzed the relationship between microstructural changes and cognitive decline after whole brain radiotherapy for brain metastases. (Mak et al. 2025) revealed the characteristics of cortical microstructural abnormalities in dementia with Lewy bodies and their association with Alzheimer's disease co-pathology. (Szeszko et al. 2025) explored white matter discriminative features of schizophrenia and schizotypic personality disorder. (Lima Santos et al. 2025) further studied the mechanism of sleep and white matter in the association between screen time and depression in children. A systematic review also highlighted the microstructural changes revealed by NODDI in multiple sclerosis (Seyedmirzaei et al., 2023). Additionally, recent studies have applied NODDI to the investigation of frontotemporal lobar degeneration (Gatto et al., 2024). Evidence has shown that NODDI is sensitive in detecting white matter and gray matter changes in subjects across the clinical spectrum of frontotemporal dementia and offers advantages over DTI, highlighting its value in the early detection and monitoring of cortical pathology (Pisano et al., 2025). These studies highlight the unique value of NODDI in unraveling brain mechanisms of neuropsychiatric disorders. The NODDI field is undergoing a shift from technology-driven to problem-driven approaches, dedicated to tackling more complex neuroscience and clinical challenges. However, large-scale, multi-center studies are needed to further establish its practical application value in disease diagnosis and treatment decision-making in order to achieve true clinical popularization of this technology.

The intersection of Mendelian randomization (MR) and neuroimaging represents a key research frontier (Figure 6B), offering a powerful tool for exploring the causal relationship between genetic factors and neuroimaging phenotypes (Guo et al., 2022; Yang et al., 2024; He et al., 2024). For example, (Zhao et al. 2023) combined MR with phenotypic data derived from white matter imaging, providing genetic evidence for the causal relationship between migraine and white matter microstructure, and offering new insights into brain structure in the context of migraine development and experience. Compared to observational studies, which are vulnerable to unmeasured confounding, MR can control confounding bias through its design. However, the causal inferences drawn from MR still require validation in additional randomized controlled trials in the future (Sekula et al., 2016).

This study has several limitations. First, while WoS and Scopus are reliable and commonly used bibliometric databases, and their separate analyses have cross-validated the findings to minimize single-database bias, relying solely on them may exclude relevant studies indexed in other databases (Ji et al., 2025; Liao et al., 2026). Second, restricting the analysis to English publications may introduce bias and limit the generalizability of the results, although the core methodological and clinical research on NODDI is predominantly published in English in international high-impact journals. Third, bibliometric analysis inherently quantifies publication volume and citation counts rather than the intrinsic scientific quality or intellectual significance of individual studies, which may lead to potential biases in cross-field comparison. Lastly, differences in algorithm design among visualization tools and manual keyword merging may introduce analytical bias, despite efforts to enhance data cleaning accuracy. Future studies should integrate multi-source data and employ mixed methods to strengthen the robustness of bibliometric conclusions.

## Conclusion

This study employed a bibliometric approach to analyze the distribution and citation patterns of publications across countries/regions, authors, and institutions. For the first time, it reveals the clear evolutionary path of the NODDI field from technical validation to diversified clinical neuroscience applications through a macro bibliometric perspective, and identifies cutting-edge interdisciplinary approaches such as Mendelian randomization as key drivers for its future growth. Additionally, the study emphasizes the importance of enhanced collaboration across regions and agencies. These findings provide insights into the current state of the field and offer a reference for researchers and organizations in identifying future research directions.

## Data Availability

The original contributions presented in the study are included in the article/Supplementary material, further inquiries can be directed to the corresponding authors.

## Supplementary material

Supplementary Figure S1 The temporal evolution of research publications in the NODDI field from the Scopus database.

Supplementary Figure S2 Keyword co-occurrence map from the Scopus database.

Supplementary Table S1 The top 20 most published journals.

Supplementary Table S2 The top 20 most published countries/regions.

Supplementary Table S3 The top 20 most cited countries/regions.

Supplementary Table S4 The top 20 most published institutions.

Supplementary Table S5 The top 10 most published authors.

Supplementary Table S6 The frequency of the top 25 keywords and the centrality.
