# Supplementary figures and images for: Bibliometric analysis of neurite orientation dispersion and density imaging: research patterns, evolution, and frontier
Source: Front Neurosci. 2026 Apr 29;20:1806164. doi: 10.3389/fnins.2026.1806164 (PMC13168155; doi:10.3389/fnins.2026.1806164)

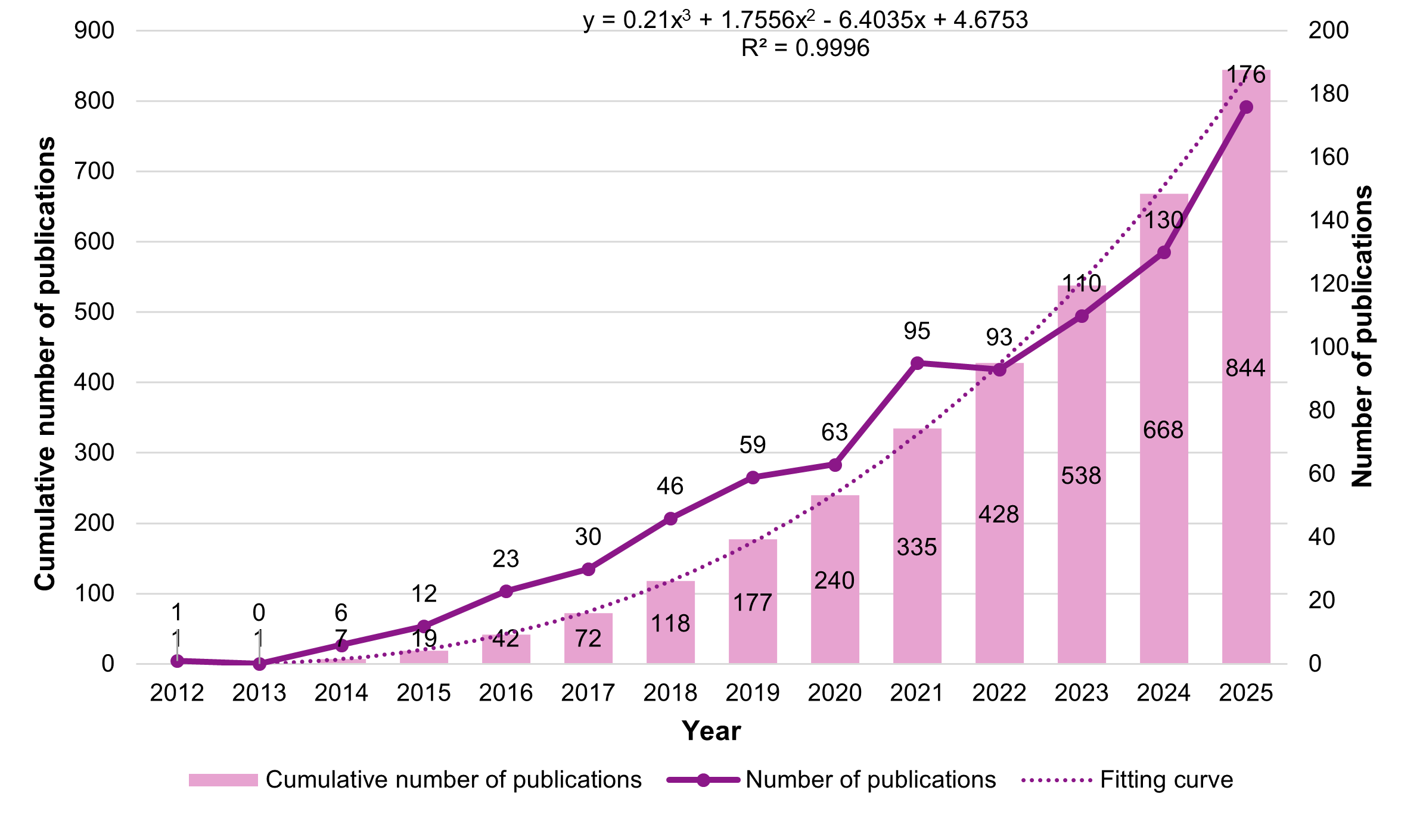

Supplement: Supplementary Figure S1 — The temporal evolution of research publications in the NODDI field from the Scopus database. [file Image_1.tif]

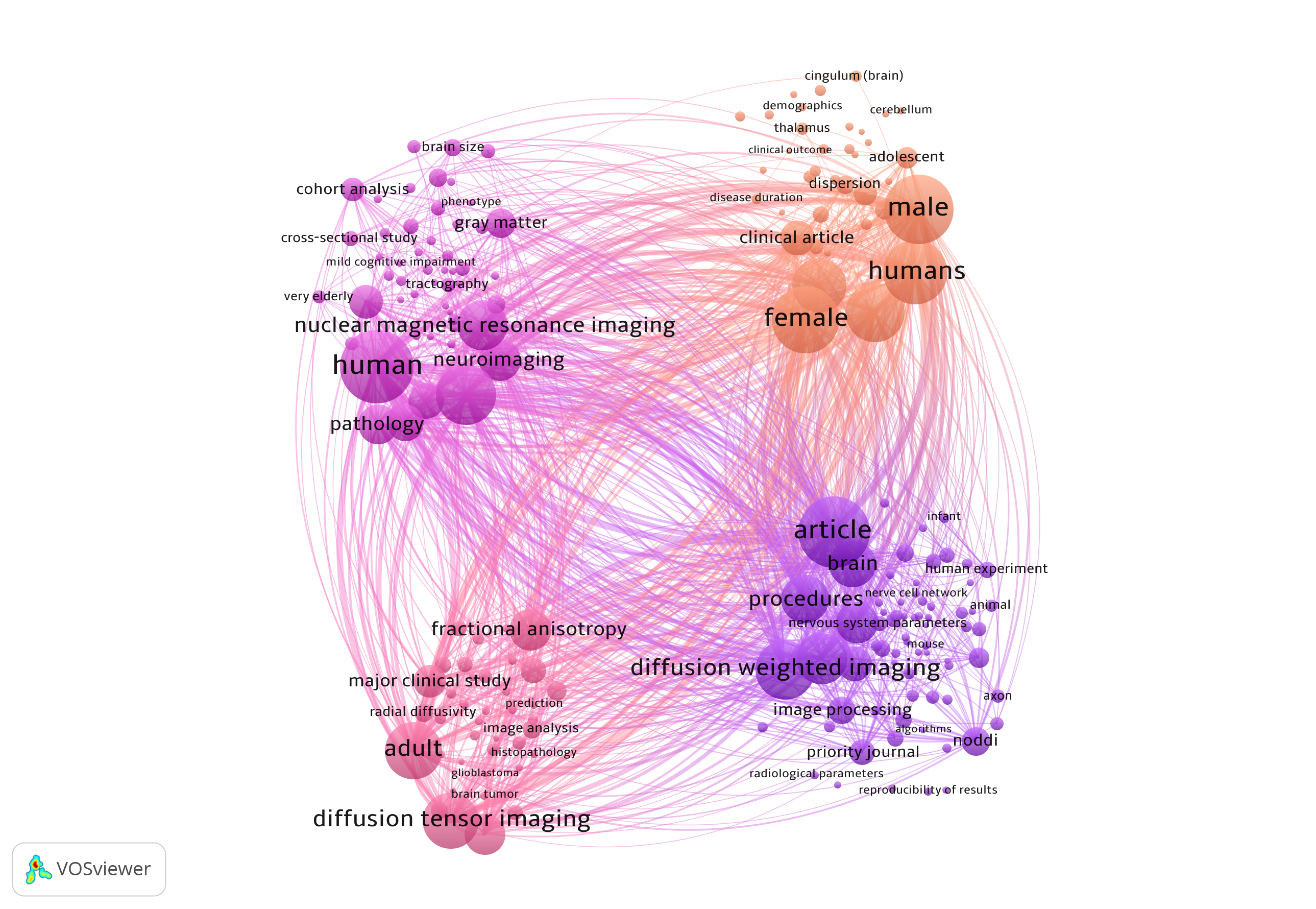

Supplement: Supplementary Figure S2 — Keyword co-occurrence map from the Scopus database. [file Image_2.tiff]
