# Supplementary material for: Bibliometric analysis of neurite orientation dispersion and density imaging: research patterns, evolution, and frontier
Source: Front Neurosci. 2026 Apr 29;20:1806164. doi: 10.3389/fnins.2026.1806164 (PMC13168155; doi:10.3389/fnins.2026.1806164)
Supplement: Supplementary Table S1 — The top 20 most published journals. [file Table_1.docx]

Table S1. The top 20 most published journals.

| **Rank** | **Journal** | **Publications** | **h-index** | **g-index** | **m-index** | **Local Citations** |
| --- | --- | --- | --- | --- | --- | --- |
| 1 | Neuroimage | 63 | 31 | 63 | 2.21 | 5585 |
| 2 | Neuroimage-Clinical | 35 | 16 | 26 | 1.33 | 744 |
| 3 | Human Brain Mapping | 26 | 13 | 26 | 1.18 | 783 |
| 4 | Frontiers in Neuroscience | 24 | 7 | 16 | 0.70 | 282 |
| 5 | Frontiers in Neurology | 22 | 9 | 14 | 1.13 | 229 |
| 6 | Magnetic Resonance in Medicine | 17 | 9 | 17 | 1.00 | 309 |
| 7 | Magnetic Resonance Imaging | 16 | 6 | 10 | 0.75 | 108 |
| 8 | Journal of Magnetic Resonance Imaging | 15 | 9 | 15 | 1.13 | 251 |
| 9 | American Journal of Neuroradiology | 11 | 6 | 11 | 0.67 | 211 |
| 10 | Cerebral Cortex | 11 | 7 | 11 | 1.17 | 242 |
| 11 | Journal of Neuroimaging | 11 | 4 | 8 | 0.40 | 66 |
| 12 | Plos One | 11 | 9 | 11 | 0.82 | 541 |
| 13 | Scientific Reports | 11 | 6 | 11 | 0.40 | 162 |
| 14 | Brain Structure & Function | 10 | 7 | 10 | 0.88 | 190 |
| 15 | Neurobiology of Aging | 10 | 7 | 10 | 0.64 | 386 |
| 16 | Neuroradiology | 10 | 5 | 10 | 0.71 | 100 |
| 17 | Brain Imaging and Behavior | 8 | 4 | 8 | 0.44 | 95 |
| 18 | Brain | 7 | 6 | 7 | 0.67 | 375 |
| 19 | Neurology | 7 | 4 | 7 | 0.50 | 135 |
| 20 | European Radiology | 7 | 4 | 7 | 0.40 | 132 |

Table S2. The top 20 most published countries/regions.

| **Rank** | **Countries/Regions** | **Publications** | **SCP** | **MCP** | **Total link strength** |
| --- | --- | --- | --- | --- | --- |
| 1 | USA | 193 | 154 | 39 | 176 |
| 2 | CHINA | 138 | 119 | 19 | 47 |
| 3 | UNITED KINGDOM | 77 | 37 | 40 | 179 |
| 4 | JAPAN | 60 | 49 | 11 | 14 |
| 5 | GERMANY | 35 | 12 | 23 | 75 |
| 6 | CANADA | 34 | 20 | 14 | 55 |
| 7 | AUSTRALIA | 22 | 10 | 12 | 36 |
| 8 | ITALY | 21 | 12 | 9 | 60 |
| 9 | FRANCE | 20 | 14 | 6 | 36 |
| 10 | NETHERLANDS | 14 | 4 | 10 | 65 |
| 11 | SWITZERLAND | 14 | 7 | 7 | 51 |
| 12 | BELGIUM | 13 | 1 | 12 | 44 |
| 13 | SPAIN | 5 | 2 | 3 | 23 |
| 14 | KOREA | 4 | 2 | 2 | 3 |
| 15 | NEW ZEALAND | 4 | 1 | 3 | 6 |
| 16 | IRAN | 3 | 3 | 0 | 1 |
| 17 | FINLAND | 2 | 1 | 1 | 4 |
| 18 | INDIA | 2 | 1 | 1 | 10 |
| 19 | NORWAY | 2 | 1 | 1 | 15 |
| 20 | PORTUGAL | 2 | 2 | 0 | 3 |

MCP, multicenter publishing; SCP, single-center publishing.

Table S3. The top 20 most cited countries/regions.

| **Rank** | **Countries/Regions** | **Total Citations** | **Average Citations** |
| --- | --- | --- | --- |
| 1 | UNITED KINGDOM | 5524 | 71.7 |
| 2 | USA | 3569 | 18.5 |
| 3 | JAPAN | 1495 | 24.9 |
| 4 | CHINA | 1142 | 8.3 |
| 5 | CANADA | 793 | 23.3 |
| 6 | SWITZERLAND | 791 | 56.5 |
| 7 | GERMANY | 727 | 20.8 |
| 8 | AUSTRALIA | 423 | 19.2 |
| 9 | FRANCE | 387 | 19.4 |
| 10 | BELGIUM | 328 | 25.2 |
| 11 | ITALY | 295 | 14 |
| 12 | NETHERLANDS | 277 | 19.8 |
| 13 | SWEDEN | 163 | 81.5 |
| 14 | NORWAY | 132 | 66 |
| 15 | SPAIN | 127 | 25.4 |
| 16 | KOREA | 60 | 15 |
| 17 | SINGAPORE | 54 | 54 |
| 18 | NEW ZEALAND | 39 | 9.8 |
| 19 | IRAN | 34 | 11.3 |
| 20 | DENMARK | 17 | 17 |

Table S4. The top 20 most published institutions.

| **Rank** | **Institutions** | **Publications** | **Centrality** |
| --- | --- | --- | --- |
| 1 | University College London | 64 | 0.16 |
| 2 | Juntendo University | 37 | 0.03 |
| 3 | University of Wisconsin System | 28 | 0.01 |
| 4 | Fujian Medical University | 20 | 0.01 |
| 5 | Harvard Medical School | 20 | 0.06 |
| 6 | King's College London | 18 | 0.07 |
| 7 | University of California San Francisco | 18 | 0.03 |
| 8 | Zhejiang University | 17 | 0.03 |
| 9 | Tokyo Metropolitan University | 16 | 0.01 |
| 10 | University of Tokyo | 16 | 0.01 |
| 11 | Capital Medical University | 14 | 0.05 |
| 12 | Toho University | 14 | 0 |
| 13 | University of Cambridge | 14 | 0.08 |
| 14 | University of Oxford | 14 | 0.06 |
| 15 | University of Toronto | 14 | 0.05 |
| 16 | University of Geneva | 13 | 0.01 |
| 17 | Vanderbilt University | 13 | 0.03 |
| 18 | Cardiff University | 12 | 0.08 |
| 19 | Murdoch Children's Research Institute | 12 | 0.03 |
| 20 | University of Edinburgh | 12 | 0.01 |

Table S5. The top 10 most published authors.

| **Rank** | **Authors** | **Publications** | **Local citations** | **h-index** | **g-index** | **m-index** | **Total link strength** |
| --- | --- | --- | --- | --- | --- | --- | --- |
| 1 | Zhang H | 43 | 1819 | 29 | 43 | 2.07 | 93 |
| 2 | Aoki S | 34 | 366 | 17 | 31 | 1.70 | 212 |
| 3 | Kamagata K | 29 | 236 | 15 | 26 | 1.50 | 186 |
| 4 | Hori M | 25 | 318 | 15 | 26 | 1.50 | 158 |
| 5 | Andica C | 24 | 140 | 13 | 23 | 1.44 | 164 |
| 6 | Alexander DC | 20 | 1437 | 18 | 20 | 1.29 | 61 |
| 7 | Uchida W | 15 | 55 | 8 | 15 | 1.33 | 119 |
| 8 | Alexander AL | 14 | 230 | 11 | 14 | 1.10 | 55 |
| 9 | Hattori N | 14 | 192 | 10 | 14 | 1.00 | 105 |
| 10 | Yu JPJ | 12 | 88 | 6 | 12 | 0.86 | 41 |

Table S6. The frequency of the top 25 keywords and the centrality.

| Rank | Keyword | Frequency | Centrality |
| --- | --- | --- | --- |
| 1 | Neurite orientation dispersion | 205 | 0.04 |
| 2 | Diffusion magnetic resonance imaging | 185 | 0.04 |
| 3 | Diffusion tensor imaging | 154 | 0.04 |
| 4 | Magnetic resonance imaging | 146 | 0.02 |
| 5 | White matter | 142 | 0.03 |
| 6 | Brain | 99 | 0.08 |
| 7 | Density | 96 | 0.04 |
| 8 | NODDI | 91 | 0.03 |
| 9 | Orientation dispersion | 81 | 0.05 |
| 10 | Diffusion | 73 | 0.01 |
| 11 | Multiple sclerosis | 69 | 0.04 |
| 12 | Microstructure | 64 | 0.02 |
| 13 | Diffusion weighted imaging | 59 | 0.11 |
| 14 | Alzheimer's disease | 58 | 0.09 |
| 15 | In vivo | 53 | 0.11 |
| 16 | Human brain | 47 | 0.04 |
| 17 | Abnormality | 45 | 0.06 |
| 18 | Model | 43 | 0.05 |
| 19 | Pathology | 43 | 0.02 |
| 20 | Gray matter | 40 | 0.02 |
| 21 | Tractography | 37 | 0.03 |
| 22 | White matter microstructure | 36 | 0.05 |
| 23 | Tensor | 35 | 0.09 |
| 24 | Connectivity | 35 | 0.05 |
| 25 | Diffusion kurtosis imaging | 33 | 0.08 |
